# Performance of MALDI-TOF Mass Spectrometry (VITEK MS) in the Identification of *Salmonella* Species

**DOI:** 10.3390/microorganisms10101974

**Published:** 2022-10-05

**Authors:** Gyu Ri Kim, Si Hyun Kim, Eun-Young Kim, Eun Hee Park, In Yeong Hwang, Seok Hoon Jeong, Hyun Soo Kim, Young Ah Kim, Young Uh, Kyeong Seob Shin, Young Ree Kim, Namhee Ryoo, Jong Hee Shin, Jeong Hwan Shin

**Affiliations:** 1 Department of Laboratory Medicine, Inje University College of Medicine, Busan 47392, Korea; 2 Department of Clinical Laboratory Science, Semyung University, Jecheon 27136, Korea; 3 Busan Institute of Health and Environment, Busan 47545, Korea; 4 Department of Laboratory Medicine and Research Institute of Bacterial Resistance, Yonsei University College of Medicine, Seoul 03722, Korea; 5 Department of Laboratory Medicine, Hallym University Dongtan Sacred Heart Hospital, Hallym University College of Medicine, Hwaseong 18450, Korea; 6 Department of Laboratory Medicine, National Health Insurance Service Ilsan Hospital, Goyang 10444, Korea; 7 Department of Laboratory Medicine, Yonsei University Wonju College of Medicine, Wonju 26411, Korea; 8 Department of Laboratory Medicine, Chungbuk National University College of Medicine, Cheongju 28644, Korea; 9 Department of Laboratory Medicine, Jeju National University Medical School, Jeju 63243, Korea; 10 Department of Laboratory Medicine, Keimyung University School of Medicine, Daegu 42601, Korea; 11 Department of Laboratory Medicine, Chonnam National University Medical School, Gwangju 61469, Korea

**Keywords:** MALDI-TOF MS, VITEK MS, mass spectrometry, *Salmonella* species

## Abstract

*Salmonella* is a major pathogen causing foodborne infections in humans. Salmonella isolates are identified using biochemical and serological tests, including automated systems such as the VITEK2 system. However, there are few reports on *Salmonella* identification using VITEK MS. Therefore, we aimed to evaluate the usefulness of MALDI-TOF VITEK MS for *Salmonella* identification. A total of 1389 *Salmonella* isolates were identified using VITEK MS ver3.0 or ver3.2. All *Salmonella* isolates were confirmed by serotyping using the Kauffmann-White scheme, and the results were compared with the VITEK MS results. A total of 1389 *Salmonella* isolates, including 66 serotypes, were correctly identified at the genus level by VITEK MS. However, these systems failed to correctly identify typhoidal *Salmonella*. Among the five *Salmonella enterica* ssp. *diarizonae* isolates, only one was correctly identified, whereas one and three isolates were partially identified and misidentified, respectively. On the other hand, the VITEK2 system successfully identified all typhoidal *Salmonella* (Typhi and Paratyphi A) and *Salmonella enterica* ssp. *diarizonae* isolates. VITEK MS was useful for identifying *Salmonella* species isolated from clinical specimens; however, additional biochemical tests, such as the VITEK2 System, should be considered to accurately identify *Salmonella* ser. Typhi, and *Salmonella* ser. Paratyphi A.

## 1. Introduction

*Salmonella* is a major pathogen causing foodborne infections, including gastroenteritis and enteric fever, in humans [1]. The genus *Salmonella* includes two species, *S. enterica* and *S. bongori*, which have similar phenotypes and genotypes [2]. *Salmonella enterica* is the most frequently isolated species of *Salmonella* and is closely associated with human infections [3].

*Salmonella* identification is routinely performed using biochemical and serological tests. Biochemical identification using automated systems, such as the VITEK2 system (bioMérieux, Lyon, France), is commonly performed in clinical microbiology laboratories [4]. Owing to advantages of speed and accuracy, matrix-assisted laser desorption ionization-time of flight mass spectrometry (MALDI-TOF MS) has recently become a routine identification system [5]. Two common MALDI-TOF MS systems, MALDI Biotyper^®^ (Bruker Daltonik GmbH, Bremen, Germany) and VITEK MS system (bioMérieux, Lyon, France), are used in clinical laboratories.

The performance of VITEK MS in identifying bacteria, fungi, and mycobacteria has been steadily evaluated [6,7,8,9,10]. However, only a few studies have evaluated the performance of VITEK MS in identifying *Salmonella* strains and serotypes [11,12,13]. This study aimed to evaluate the usefulness of MALDI-TOF VITEK MS for *Salmonella* identification using more than 1000 *Salmonella* strains, including 60 serotypes, isolated from human specimens.

## 2. Materials and Methods

### 2.1. Clinical Isolates

In total, 1389 *Salmonella* strains isolated from various human specimens such as stool, blood, urine, body fluids, and tissues were included in this study.

### 2.2. Final Identification by Serotyping

*Salmonella* strains were identified by serotyping using the White-Kauffmann-Scheme with the slide agglutination test for somatic antigens and the tube agglutination test for flagella antigens, as in our previous report [14,15].

### 2.3. Identification by VITEK MS and VITEK2 Systems

All *Salmonella* strains were identified using the VITEK MS system (bioMérieux, Lyon, France), according to the manufacturer’s instructions. The results were interpreted using VITEK MS v3.0, (*n* = 1167) until October 2019, and VITEK MS v3.2 (*n* = 222) was used thereafter. A fresh colony was smeared onto a 48-wall target plate and covered with 1 µL of α-cyano-4-hydroxycinnamic acid (CHCA) matrix solution. After drying, the target plate was loaded into the MALDI-TOF VITEK MS system [4]. The developed MS fingerprint was automatically compared to the VITEK MS database v3.0 and v3.2. *Escherichia coli* ATCC 8739 was used as the quality control strain. Additionally, we tested the VITEK2 system for 93 typhoidal *Salmonella*, including 20 *Salmonella* ser. Paratyphi A, 9 *Salmonella* ser. Paratyphi B, 64 *Salmonella* ser. Typhi, and 5 *Salmonella enterica* subsp. *diarizonae*.

### 2.4. Database and Analysis

Table 1 shows the database of the VITEK MS ver3.0, ver3.2, and VITEK2 systems for *Salmonella* spp. VITEK MS v3.0 reports the results for *Salmonella* ser. Typhi, *Salmonella* ser. Paratyphi A, *Salmonella* ser. Gallinarum, *Salmonella enterica* subsp. *arizonae*/*Salmonella enterica* subsp. *diarizonae*, and *Salmonella* groups. The *Salmonella* group refers to all *S. enterica* strains other than those mentioned above. The reports of VITEK MS v3.2 were simplified as two results: *Salmonella enterica* ssp. *arizonae*/*Salmonella enterica* ssp. *diarizonae* and *Salmonella enterica* ssp. *enterica.* For the VITEK2 system, six results were included in the database, including the *Salmonella* ser. Typhi, *Salmonella* ser. Paratyphi A, *Salmonella* ser. Gallinarum, *Salmonella enterica* subsp. *arizonae*, *Salmonella enterica* subsp. *diarizonae*, and *Salmonella* groups.

The results of the VITEK MS were compared with those of the final identification by serotyping. The difference in the results between the VITEK2 system and VITEK MS for 93 typhoidal *Salmonella* and 5 *Salmonella enterica* ssp. *diarizonae* were analyzed.

## 3. Results

A total of 1389 *Salmonella* strains were included in this study (Table 2). These comprised 66 serotypes of 1167 *Salmonella* isolates for VITEK MS v3.0 analysis, and 27 serotypes of 222 *Salmonella* isolates for VITEK MS v3.2. In the analysis using VITEK MS v3.0, 72 typhoidal *Salmonella* spp. (14 *Salmonella* serovar. Paratyphi A, 7 *Salmonella* ser. Paratyphi B, 51 *Salmonella* ser. Typhi), 2 *Salmonella enterica* subsp. *diarizonae* (1 *Salmonella* ser. IIIb 47:r:z and 1 *Salmonella* ser. IIIb 48:k:z), and 1093 other *Salmonella enterica* subsp. *enterica* were included. In the analysis using VITEK MS v3.2, 21 typhoidal *Salmonella* isolates (6 *Salmonella* ser. Paratyphi A, 2 *Salmonella* serovars. Paratyphi B, and 13 *Salmonella* ser. Typhi), 3 *Salmonella enterica* subsp. *diarizonae* (1 *Salmonella* ser. IIIb 47:r:z and 2 *Salmonella* ser. IIIb 48:k:z), and 198 other *Salmonella enterica* ssp. *enterica* were included.

All 1389 *Salmonella* strains were correctly identified as *Salmonella* by VITEK MS at the genus level (Table 3). The results of VITEK MS v3.0 (*n* = 1167) were as follows: *Salmonella* group (*n* = 1157), *Salmonella* ser. Paratyphi A (*n* = 3), *Salmonella* ser. Paratyphi A/Salmonella (*n* = 3), *Salmonella* ser. Typhi/*Salmonella* group (*n* = 3), and *Salmonella enterica* ssp. *arizonae/Salmonella enterica* ssp. *diarizonae* (*n* = 1). The results of VITEK MS v3.2 (*n* = 222) were as follows: *Salmonella enterica* ssp. *enterica* (*n* = 221), and *Salmonella enterica* ssp. *enterica/S. enterica* ssp. *arizonae/S. enterica* ssp. *diarizonae* (*n* = 1).

We compared the results of VITEK MS v3.0 and 3.2 with those of serotyping and the VITEK2 system for 98 *Salmonella* isolates (Table 4). VITEK MS v3.0. did not correctly identify typhoidal *Salmonella* and *Salmonella enterica* ssp. *diarizonae*, although these were included in the ver3.0. Only 3 of the 14 *Salmonella* ser. Paratyphi A isolates were correctly identified, and the other three isolates were partially identified as *Salmonella* ser. Paratyphi A/Salmonella group. The remaining eight isolates were assigned to the *Salmonella* group. Most of the *Salmonella* ser. Typhi was reported to belong to the *Salmonella* group, although three isolates were partially identified as *Salmonella* ser. Typhi/Salmonella group. For two *Salmonella enterica* ssp. *diarizonae* isolates, *Salmonella* ser. IIIb 47:r:z was reported as *Salmonella* group and *Salmonella* ser. IIIb 48:k:z was reported as *Salmonella enterica* subsp. *arizonae/Salmonella enterica* subsp. *diarizonae*. However, all *Salmonella* ser. Paratyphi A, *Salmonella* ser. Typhi, and *Salmonella enterica* ssp. *diarizonae* were correctly identified using the VITEK2 system.

There were a few changes in the VITEK MS v3.2 as described in the Methods. VITEK MS v3.2 system identified all six *Salmonella* ser. Paratyphi A and 13 *Salmonella* ser. Typhi as *Salmonella enterica* subsp. *enterica*. Two *Salmonella enterica* ssp. *diarizonae*, including one *Salmonella* ser. IIIb 47:r:z and one *Salmonella* ser. IIIb 48:k:z were misidentified as *Salmonella enterica* subsp. *enterica*. The remaining one *Salmonella enterica* ssp. *diarizonae* was partially identified as *Salmonella enterica* ssp. *enterica/Salmonella enterica* ssp. *arizonae/Salmonella enterica* ssp. *diarizonae*. All *Salmonella* isolates were correctly identified using the VITEK2 system.

## 4. Discussion

More than 2600 serotypes of *Salmonella*, including 46 type O serogroups and 114 type H serogroups, have been reported [2]. The two major antigens that determine the serotype are bacterial somatic antigens corresponding to O-polysaccharide and flagella antigens corresponding to flagellin proteins. Accurate serotype identification is crucial because the virulence of *Salmonella*, especially typhoidal *Salmonella*, varies depending on the serotype [15].

*Salmonella* identification has been performed using biochemical and serological tests. Automated identification systems such as the VITEK2 system provide rapid, reliable, and highly reproducible results [16]. Recently, MALDI-TOF MS has been commonly used as a routine identification method [5,6]. However, there are a few previous reports on the performance of VITEK MS in identifying *Salmonella* [11,12,13]. Guo et al. [17] using 1025 bacteria isolated from clinical specimens, reported that VITEK MS exhibited good performance; however, only two strains of *Salmonella* were included in the report. Richter et al. [6] reported that VITEK MS exhibited good performance in identifying *Enterobacteriaceae* at the genus and species levels. They reported that 35 isolates of *Salmonella enterica* ssp. *enterica* were included, and 33 and 2 strains were correctly identified at the species and genus levels, respectively, using VITEK MS v2.0. Therefore, they concluded that VITEK MS would be appropriate for the identification of *Salmonella* at the genus and species levels with high accuracy. Wattal et al. [18] evaluated VITEK MS using 12,003 microbial isolates, including *Enterobacterales*, other gram-negative bacteria, gram-positive bacteria, yeast, fungi, and mycobacteria. They reported that VITEK MS correctly identified 95.8% of the isolates at the species level. Among the 145 *Salmonella* isolates, 138 isolates were identified as *Salmonella*; however, seven *Salmonella* ser. Typhi isolates showed no identification results. Interestingly, they reported that all 18 *Salmonella* spp. Paratyphi A and 44/51 *Salmonella* ser. Typhi were correctly identified at the serotype level. In our study, we evaluated the performance of VITEK MS with numerous *Salmonella* isolates consisting of 66 confirmed serotypes, and all 1389 *Salmonella* isolates were correctly identified as *Salmonella*, consistent with the above studies. Collectively, these findings demonstrate that VITEK MS is suitable for the identification of *Salmonella* at the genus level.

In our study, only three *Salmonella* ser. Paratyphi A isolates were correctly identified using the VITEK MS v3.0 among 14 *Salmonella* ser. Paratyphi A and 51 *Salmonella* ser. Typhi isolates, which is in contrast with the above report by Wattal et al. [18]. Moreover, three *Salmonella* ser. Paratyphi A and three *Salmonella* ser. Typhi isolates were partially identified, whereas the others were identified as *Salmonella* group using VITEK MS v3.0. In the updated VITEK MS v3.2, the serotypes of *Salmonella* ser. Paratyphi A, *Salmonella* ser. Typhi, and *Salmonella* ser. Gallinarum were removed from the database. Another MALDI-TOF MS device, MALDI Biotyper^®^ (Bruker Daltonik GmbH, Germany) is also widely used in clinical laboratories. Bastin et al. [19] reported that the MALDI Biotyper could identify 100% of *Salmonella* isolates at the genus level; however, it failed to correctly identify the serotype for typhoidal *Salmonella*.

Using the VITEK2 system, *Salmonella* ser. Typhi isolates were correctly identified at the serotype level. Therefore, additional biochemical tests, such as the VITEK2 system should be performed for the accurate identification of *Salmonella* ser. Paratyphi A and *Salmonella* ser. Typhi. Nevertheless, *Salmonella* ser. Paratyphi B cannot be correctly identified at the serotype level by either the VITEK2 system or VITEK MS, and additional tests are needed.

There are few reports on the identification of *Salmonella enterica* ssp. *arizonae/Salmonella enterica* ssp. *diarizonae* using VITEK MS. In this study, we found that the results for *Salmonella enterica* ssp. *arizonae/S. enterica* ssp. *diarizonae* using VITEK MS v3.0 and v3.2 were not suitable for the final identification of the pathogens (Table 4). Only one *Salmonella enterica* ssp. *diarizonae* isolate was correctly identified by VITEK MS. In contrast, one and three *Salmonella enterica* ssp. *diarizonae* isolates were partially identified and misidentified, respectively. Nevertheless, these five isolates were correctly identified as *Salmonella enterica* ssp. *diarizonae* using the VITEK2 system. Therefore, we believe that biochemical identification systems, such as the VITEK2 system, are useful for the accurate identification of *Salmonella enterica* subsp. *diarizonae*.

## 5. Conclusions

In this study, we demonstrated that VITEK MS can identify most of the common serotypes of *Salmonella* in the *Salmonella* group or *Salmonella enterica* subsp. *enterica* with 100% sensitivity. However, additional tests, such as the VITEK2 system, are required to confirm the presence of typhoidal *Salmonella* spp. *(Salmonella* ser. Typhi, and *Salmonella* ser. Paratyphi A).

## Figures and Tables

**Table 1:** Database of VITEK MS v3.0, v3.2, and VITEK2 system for *Salmonella*.

System Type	Database List
VITEK2 system	*Salmonella* group
	*Salmonella* ser. Gallinarum
	*Salmonella* ser. Paratyphi A
	*Salmonella* ser. Typhi
	*Salmonella enterica* ssp. *arizonae*
	*Salmonella enterica* ssp. *diarizonae*
VITEK MS v3.0	*Salmonella* group
	*Salmonella* ser. Gallinarum
	*Salmonella* ser. Paratyphi A
	*Salmonella* ser. Typhi
	*Salmonella enterica* ssp. *arizonae/diarizonae*
VITEK MS v3.2	*Salmonella enterica* ssp. *enterica*
	*Salmonella enterica* ssp. *arizonae/diarizonae*

**Table 2:** Serotype distribution of *Salmonella* in this study.

Serotype	VITEK MS v3.0	VITEK MS v3.2
	(*n* = 1167)	(*n* = 222)
	N	N
I4,[5],12:i:-	220	38
Enteritidis	171	65
Bareilly	121	21
Typhimurium	87	10
Infantis	83	22
Thompson	56	1
Agona	53	6
Typhi	51	13
Montevideo	34	4
Livingstone	34	0
Stanley	26	1
Virchow	20	2
Panama	20	2
Newport	16	6
Saintpaul	15	2
Paratyphi A	14	6
Mbandaka	14	1
Braenderup	11	4
Othmarschen	10	3
Rissen	9	3
Paratyphi B	7	2
Others †	95	10

† Others: VITEK MS v3.0: *Salmonella* ser. Agama, Agbeni, Albany, Bovismorbificans, Brunei, Cerro, Choleraesuis, Derby, Dessau, Ebrie, Essen, Give, Hadar, Hato, Heidelberg, Hindmarsh, I4,[5],12:-:-, Inganda, Kentucky, Kingston, Konstanz, Kottbus, Litchfield, London, Muenchen, Muenster, Ohio, Oslo, Ponoma, Poona, Reading, Sandiego, Schleissheim, Schwarzemgrund, Senftenberg, Simi, Singapore, Sinstorf, Uganda, Urbana, Wa, Weltevreden, Weltevreden var. 15+, IIIb 47:r:z, and IIIb 48:k:z. VITEK MS v3.2: *Salmonella* ser. Derby, Give, London, Ohio, Simi, IIIb 47:r:z, IIIb 48:k: z.

**Table 3:** *Salmonella* identification results by VITEK MS v3.0 and v3.2.

VITEK MS (N)	MALDI-TOF VITEK MS Results	*n* (%)
v3.0 (1167)	*Salmonella* group	1157 (99.1)
	*Salmonella* ser. Paratyphi A	3 (0.3)
	*Salmonella* ser. Paratyphi A/*Salmonella* group	3 (0.3)
	*Salmonella* ser. Typhi/*Salmonella* group	3 (0.3)
	*Salmonella enterica* ssp. *arizonae*/*Salmonella enterica* ssp. *diarizonae*	1 (0.1)
v3.2 (222)	*Salmonella enterica* ssp. *enterica*	221 (99.5)
	*Salmonella enterica* ssp. *enterica*/*Salmonella enterica* ssp. *arizonae*/*Salmonella enterica* ssp. *diarizonae*	1 (0.5)

**Table 4:** Comparison between the performance of VITEK MS and VITEK2 systems in identifying serotype or subspecies.

VITEK MS	Serotype (*n*)	N	VITEK MSs	VITEK2 System
v3.0	Paratyphi A (14)	8	*Salmonella* group	*Salmonella* ser. Paratyphi A
		3	*Salmonella* ser. Paratyphi A	*Salmonella* ser. Paratyphi A
		3	*Salmonella* ser. Paratyphi A / *Salmonella* group	*Salmonella* ser. Paratyphi A
	Paratyphi B (7)	7	*Salmonella* group	*Salmonella* group
	Typhi (51)	48	*Salmonella* group	*Salmonella* ser. Typhi
		3	*Salmonella* ser. Typhi *Salmonella* group	*Salmonella* ser. Typhi
	IIIb 47:r:z (1)	1	*Salmonella* group	*Salmonella enterica* ssp. *diarizonae*
	IIIb 48:k:z (1)	1	*Salmonella enterica* ssp. *arizonae*/*Salmonella enterica* ssp. *diarizonae*	*Salmonella enterica* ssp. *diarizonae*
v3.2	Paratyphi A (6)	6	*Salmonella enterica* ssp. *enterica*	*Salmonella* ser. Paratyphi A
	Paratyphi B (2)	2	*Salmonella enterica* ssp. *enterica*	*Salmonella* group
	Typhi (13)	13	*Salmonella enterica* ssp. *enterica*	*Salmonella* ser. Typhi
	IIIb 47:r:z (1)	1	*Salmonella enterica* ssp. *enterica*	*Salmonella enterica* ssp. *diarizonae*
	IIIb 48:k:z (2)	1	*Salmonella enterica* ssp. *enterica*	*Salmonella enterica* ssp. *diarizonae*
		1	*Salmonella enterica* ssp. *enterica*/*Salmonella enterica* ssp. *arizonae*/*Salmonella enterica* ssp. *diarizonae*	*Salmonella enterica* ssp. *diarizonae*

## Data Availability

Patient consent was waived because personal information was not used.
